# Ketamine and Esketamine in Neurology and Psychiatry: An Overview

**DOI:** 10.7759/cureus.108571

**Published:** 2026-05-09

**Authors:** Amalesh S Honnekeri

**Affiliations:** 1 General Medical Practice, Honnekeri Clinic, Mumbai, IND

**Keywords:** esketamine, interventional psychiatry, ketamine, major depressive disorder, neuropsychiatry, treatment-resistant depression

## Abstract

Treatment-resistant depression (TRD) remains a significant clinical concern, particularly in patients who do not achieve adequate response with conventional antidepressant therapies. Ketamine and esketamine, which act on glutamatergic pathways, have been introduced into clinical practice as alternative therapeutic approaches within interventional psychiatry. This narrative review outlines key aspects of ketamine and esketamine use in psychiatric settings, including proposed mechanisms of action, clinical use cases, methods of administration, safety profiles, and areas of ongoing discussion. Ketamine is generally administered intravenously in monitored clinical settings, while esketamine is available as an intranasal formulation used under supervision. Considerations related to tolerability and monitoring requirements are relevant in clinical use. Ketamine and esketamine represent a developing area in the treatment of TRD and highlight the broader emergence of interventional approaches in psychiatry. Additional research is warranted to further characterize long-term outcomes, refine treatment protocols, and better define patient selection criteria.

## Introduction and background

Depression is known to be a leading cause of disability worldwide, often resulting in mortality [1]. Major depressive disorder (MDD) has a multifactorial etiology, with biology, genetics, the patient’s surrounding environment, and psychosocial factors playing causative roles [1]. MDD has a lifetime prevalence ranging from 5% to 17%, with an average of 12% [1]. Women are twice as likely to suffer from MDD as men [1].

It is imperative to note that gamma-aminobutyric acid (GABA), an inhibitory neurotransmitter, is implicated in the development of depression [1]. Its levels have been found to be decreased in the plasma, cerebrospinal fluid, and brain in patients suffering from depression [1].

Studies have demonstrated that nearly 40% of patients do not respond appropriately to conventional treatment and are classified as ‘treatment-resistant’ (patients who have not shown a clinical response to at least two consecutive antidepressant trials that have been prescribed at adequate doses for a minimum of four to six weeks) [2-4]. Treatment-resistant depression (TRD) poses a major healthcare challenge, leading to negative patient outcomes such as increased morbidity and mortality, as well as increased healthcare costs [3,4].

Traditional antidepressants primarily target monoaminergic neurotransmitter systems, including the serotonergic, noradrenergic, and dopaminergic pathways, which play a central role in regulating mood, emotional processing, and cognitive function. These systems arise from brainstem nuclei (principally the raphe nuclei, locus coeruleus, and ventral tegmental area) and project extensively to cortical and limbic structures, such as the prefrontal cortex, hippocampus, and amygdala [4-7]. Antidepressant classes, including selective serotonin reuptake inhibitors (SSRIs), serotonin-norepinephrine reuptake inhibitors (SNRIs), tricyclic antidepressants (TCAs), and monoamine oxidase (MAO) inhibitors, act by increasing synaptic concentrations of monoamines via inhibition of their reuptake or enzymatic breakdown [5-7]. Although these effects occur rapidly at the synaptic level, therapeutic benefit is typically delayed, likely due to downstream processes, such as receptor desensitization, modulation of intracellular signaling pathways, and changes in gene expression within these neural circuits [5-7]. Thus, traditional antidepressants often require several weeks to exert therapeutic effects [5]. Unfortunately, during this ‘lag period’ before which they exert their full effects, the patient continues to suffer from their symptoms, putting themselves as well as their personal and professional lives at risk of harm [5]. This is therefore considered to be a major limitation, resulting in the risk of suicidality, especially in the first nine days after starting antidepressant treatment [6].

Ketamine, a non-competitive N-methyl-D-aspartate (NMDA) receptor (NMDAR) antagonist originally developed as an anesthetic, has emerged as a rapid-acting antidepressant with robust effects observed within hours of administration [5]. Initial investigations by Skolnick et al. demonstrated that NMDA antagonists improved depressive symptoms in animal models [8]. Subsequent studies confirmed that ketamine, in particular, had antidepressant effects in animal models [7-9]. Further human studies have confirmed ketamine’s rapid antidepressant effects in humans [5,10].

In 2019, the Food and Drug Administration (FDA) approved intranasally administered esketamine (the S-enantiomer of ketamine) for TRD in adults [11]. In 2020, it also received FDA approval for the treatment of adults with MDD and acute suicidal ideation or behavior [11].

Despite the growing body of literature on ketamine and esketamine, several important gaps remain. Existing studies vary widely in design, dosing protocols, and outcome measures, and there is limited consensus regarding optimal treatment strategies, long-term safety, and maintenance regimens. Furthermore, evidence from real-world clinical settings and diverse patient populations remains insufficient, and the integration of these agents into routine psychiatric practice continues to evolve.

In this context, a comprehensive synthesis of current evidence is necessary to consolidate existing knowledge, highlight areas of uncertainty, and inform clinical decision-making. This narrative review aimed to summarize the current evidence regarding ketamine and esketamine in neuropsychiatry, with particular emphasis on pharmacology, clinical efficacy, administration protocols, safety considerations, and future directions.

## Review

Pharmacology and mechanism of action

A review conducted by Zanos and Gould compiled various hypotheses pertaining to the mechanism of action of ketamine as a rapid-acting antidepressant drug [12]. Unlike traditional antidepressants, ketamine and esketamine do not rely primarily on serotonin, norepinephrine, or dopamine modulation, representing a distinct therapeutic mechanism [12].

NMDARs are glutamatergic, ligand-gated ion channel receptors that exist structurally as heterotetramers. To date, seven subtypes of NMDARs have been identified: GluN1, GluN2A, GluN2B, GluN2C, GluN2D, GluN3A, and GluN3B [13].

Disinhibition Hypothesis

According to this hypothesis, NMDARs on GABAergic inhibitory interneurons are selectively blocked by ketamine. This causes a disinhibition of pyramidal neurons and enhanced glutamatergic firing. The glutamate that is released from this evoked firing binds to post-synaptic α-amino-3-hydroxy-5-methyl-4-isoxazolepropionic acid receptors (AMPARs), causing their activation. This results in enhanced brain-derived neurotrophic factor (BDNF) release, activation of the tropomyosin receptor kinase B (TrkB) receptor, and subsequently, promotion of protein synthesis via activation of the mechanistic target of rapamycin complex 1 (mTORC1) [12-14]. BDNF is thought to be an instructive mediator of neuroplasticity, influencing dendritic spines and, within the hippocampus, adult neurogenesis [15]. Changes in the rate of adult neurogenesis and in spine density can influence several forms of learning and memory, as well as contribute to depression [15]. mTORC1 acts as the central regulator of cellular functions [16]. AMPARs are important ionotropic glutamate receptors that mediate the majority of fast excitatory synaptic transmission in the central nervous system; their activation is required for the fast-acting antidepressant effects of ketamine [17]. Ketamine is an enantiomeric mixture of R-ketamine and S-ketamine. S-ketamine has an approximately four-fold greater potency at inhibiting NMDAR compared to its R-ketamine enantiomer [18].

Inhibition of Extrasynaptic NMDARs

Per this theory, ketamine selectively blocks extrasynaptic GluN2B-containing NMDARs. These NMDARs are tonically activated by low levels of ambient glutamate, which is regulated by the glutamate transporter 1 located on astrocytes. The inhibition of these NMDARs is thought to desuppress mTORC1 function, which in turn induces protein synthesis [12].

Blockade of Spontaneous Activation of NMDARs

According to this hypothesis, ketamine blocks the spontaneous activation of NMDARs, which mediates spontaneous neurotransmission. This results in the inhibition of the eukaryotic elongation factor 2 (eEF2) kinase (eEF2K) activity, thereby preventing phosphorylation of its eEF2 substrate. This effect subsequently leads to an enhancement of BDNF translation [12].

Role of Metabolite of Ketamine-Hydroxynorketamine (HNK)

Per this hypothesis, ketamine, via its metabolites, (2R,6R)-HNK and (2S,6S)-HNK, exhibits NMDAR inhibition-independent antidepressant actions. On administration, ketamine is metabolized to HNKs, which promote AMPAR-mediated synaptic potentiation [12].

These pathways promote synaptogenesis and enhanced neuroplasticity, which are believed to account for the rapid antidepressant effects of ketamine and esketamine [12]. Unlike traditional monoaminergic antidepressants, which exert their effects indirectly through gradual modulation of serotonin, norepinephrine, and downstream receptor adaptations over several weeks, ketamine acts by directly enhancing glutamatergic transmission and activating intracellular signaling pathways such as BDNF and mTORC1; this leads to rapid synaptic remodeling and restoration of functional connectivity in key mood-regulating circuits, thereby explaining the markedly faster onset of antidepressant effects observed with ketamine and esketamine [4-7,12].

While these hypotheses differ in their proximal mechanisms, they are not mutually exclusive and likely represent complementary pathways contributing to ketamine’s antidepressant effects. A unifying feature across these models is the enhancement of glutamatergic signaling through AMPAR activation and the subsequent engagement of intracellular cascades involving BDNF and mTORC1. These convergent pathways promote synaptogenesis and neuroplasticity, ultimately restoring functional connectivity in key mood-regulating circuits. Thus, rather than acting through a single mechanism, ketamine appears to exert its rapid antidepressant effects through a coordinated network of interrelated processes that converge on synaptic strengthening and neural circuit remodeling.

**Figure 1:**
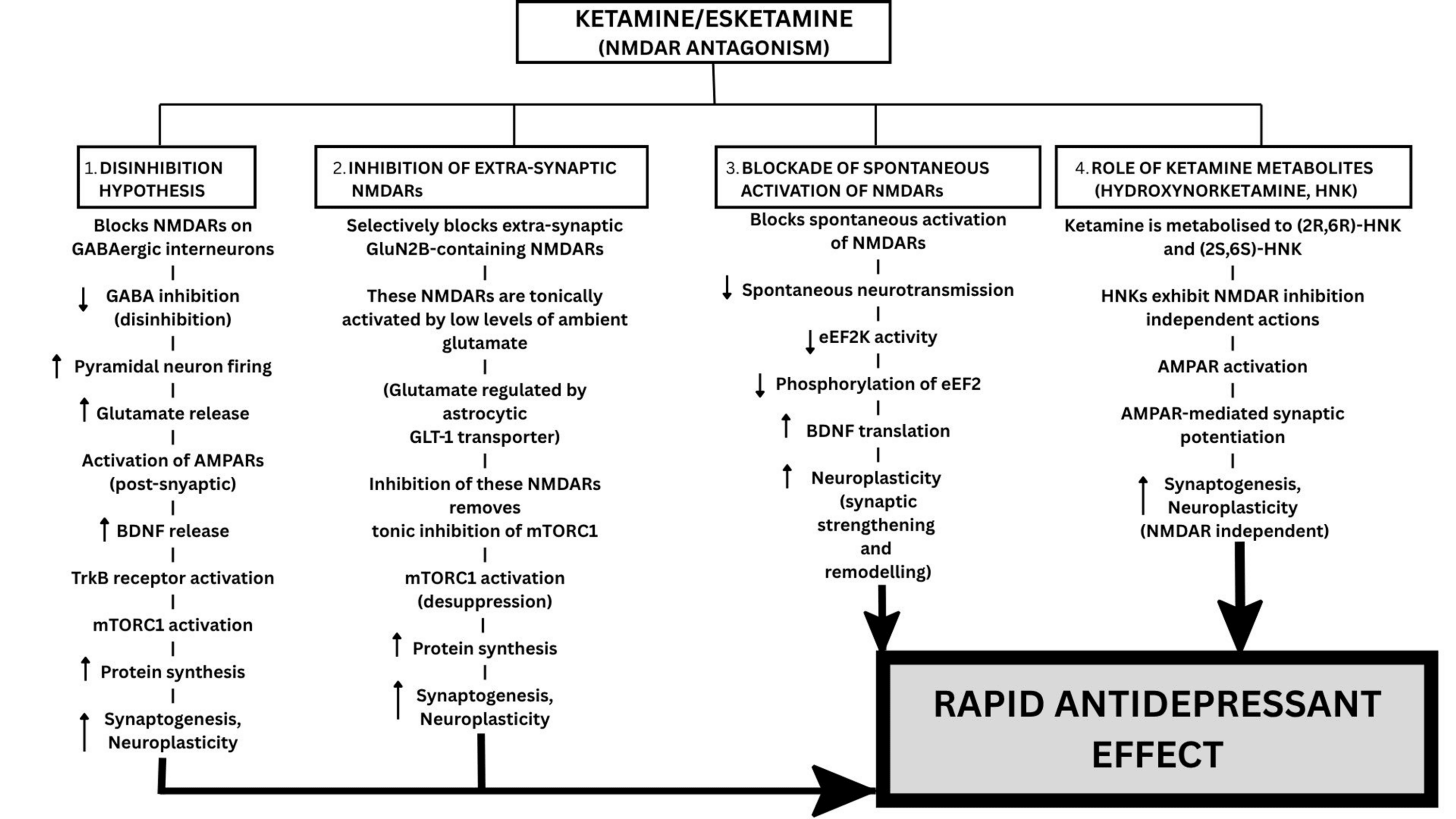
Schematic representation of the proposed mechanisms underlying the antidepressant effects of ketamine and esketamine NMDAR: N-methyl-D-aspartate receptor; AMPAR: α-amino-3-hydroxy-5-methyl-4-isoxazolepropionic acid receptor; BDNF: brain-derived neurotrophic factor; TrkB: tropomyosin receptor kinase B; mTORC1: mechanistic target of rapamycin complex 1; eEF2K: eukaryotic elongation factor 2 kinase; eEF2: eukaryotic elongation factor 2; GABA: gamma-aminobutyric acid; HNK: hydroxynorketamine The figure was created by the author based on information from [12], using Canva (Canva Pty Ltd., Sydney, Australia).

Figure 1 presents a flow chart that schematically represents the proposed mechanisms underlying the antidepressant effects of ketamine and esketamine [12].

**Figure 2:**
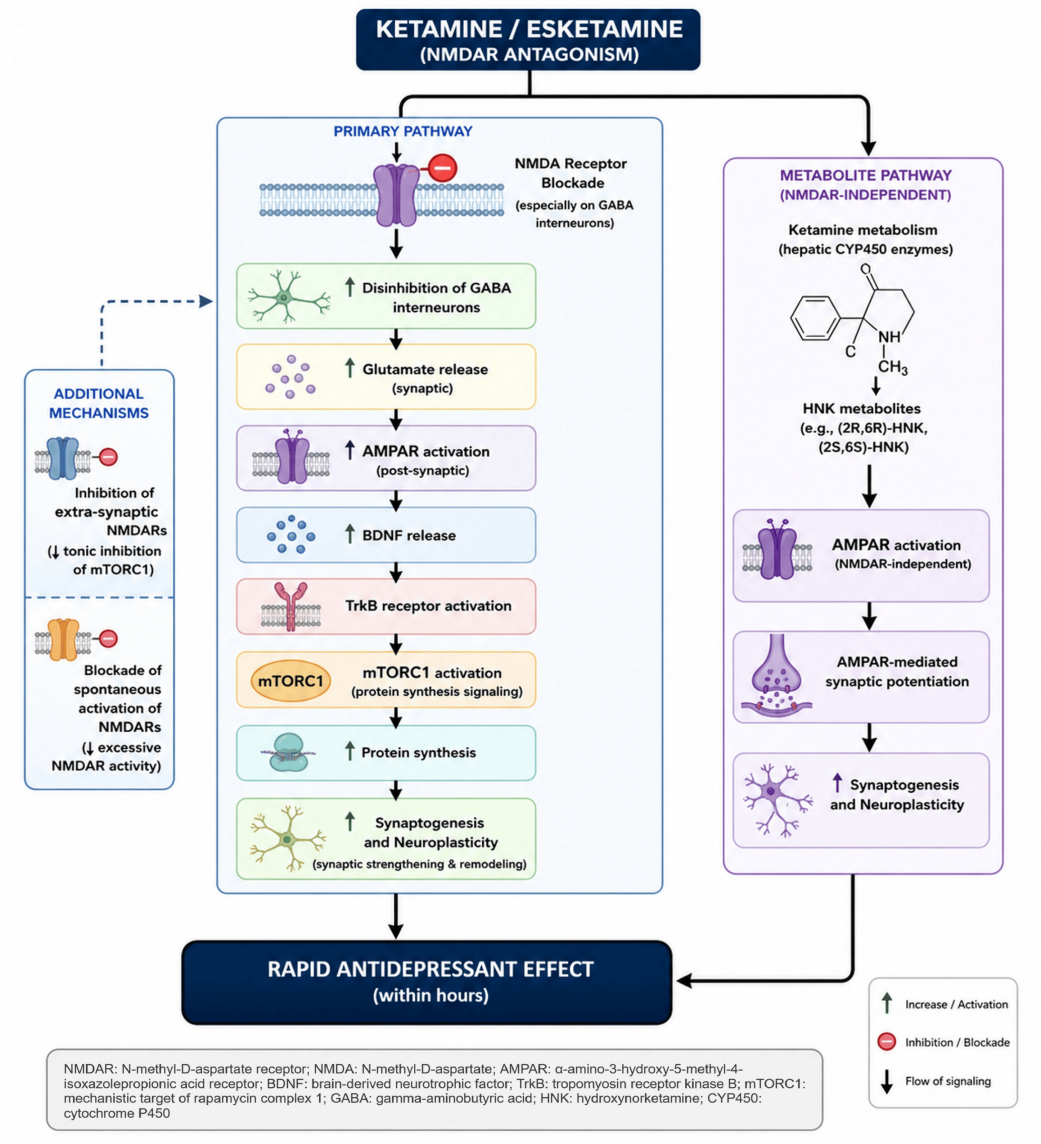
Pictographic representation of the proposed mechanisms underlying the antidepressant effects of ketamine and esketamine The figure was created by the author based on information from [12], using Canva (Canva Pty Ltd., Sydney, Australia) and ChatGPT (OpenAI, San Francisco, USA).

Figure 2 presents a pictographic representation of the proposed mechanisms underlying the antidepressant effects of ketamine and esketamine [12].

While the antidepressant effects of ketamine have traditionally been attributed to NMDAR antagonism and downstream enhancement of glutamatergic signaling, emerging evidence suggests additional modulatory pathways may also contribute [19]. Recent findings indicate that ketamine may reduce neuronally released glutamate via a retrograde signaling mechanism involving presynaptic adenosine A1 receptors. In this model, ketamine-induced increases in extracellular adenosine lead to activation of A1 receptors, which in turn suppress presynaptic glutamate release, thereby modulating excitatory neurotransmission [19]. This mechanism highlights a potential role for purinergic signaling in mediating the complex and bidirectional effects of ketamine on glutamatergic neurotransmission.

Clinical applications of ketamine and esketamine in neuropsychiatry

In patients suffering from depression, a 40-minute intravenous (IV) infusion of ketamine (commonly administered as a racemic mixture containing equal parts of R-ketamine and S-ketamine enantiomers) at doses of 0.2-0.5 mg/kg has demonstrated antidepressant responses within two hours of administration, with effects sustained for several days and, in some cases, up to two weeks following a single dose [20-23]. Esketamine, the S-enantiomer of ketamine, has a higher affinity for NMDAR and is the formulation used in intranasal preparations approved for clinical use [23]. Intranasal administration of esketamine at doses of 28-84 mg twice weekly for a period of two weeks has demonstrated significant antidepressant effects in patients with TRD when used as an adjunct to oral antidepressant therapy [21,24].

TRD

The strongest evidence for the use of ketamine and esketamine lies in their role in TRD. IV ketamine administered at 0.5 mg/kg over 40 minutes has demonstrated significant reductions in depressive symptoms within hours of treatment [22].

A meta-analysis by Bahji et al. included 36 randomized controlled trials with 2,903 participants, of which nine trials evaluated esketamine and the remaining studies evaluated racemic ketamine. Ketamine or esketamine treatment was associated with significantly higher response rates (risk ratio (RR) = 2.14; 95% CI: 1.72 to 2.66), remission rates (RR = 1.64; 95% CI: 1.33 to 2.02), and greater improvement in depression severity (Cohen’s d = -0.63; 95% CI: -0.80 to -0.45) compared with placebo [23]. The aforementioned meta-analysis did not, however, find any difference in efficacy on the administration of a single dose compared to repeat doses. In maintenance studies, esketamine was also found to be associated with lower relapse rates compared with placebo [23]. This meta-analysis reiterated that ketamine exerts its effects by antagonizing NMDARs and enhancing AMPAR-mediated glutamatergic transmission, which rapidly activates intracellular second messenger pathways, including BDNF release and mTORC1 signaling, leading to synaptic protein synthesis and increased dendritic spine formation [23]. Unlike the gradual neuroadaptive changes seen with traditional antidepressants, these processes can occur within hours, resulting in rapid functional restoration of synaptic connectivity in key mood-regulating circuits and thereby explaining the early onset of antidepressant effects, with sustained benefits attributed to longer-term neuroplastic changes [5-7,23]. 

Although subgroup analyses suggested numerically stronger effects for racemic ketamine, esketamine has a stronger regulatory evidence base for TRD, including larger phase III trials and relapse-prevention data. In a randomized relapse-prevention trial, continued esketamine plus oral antidepressant reduced relapse risk by 51% in patients with stable remission and 70% in those with stable response compared with oral antidepressant plus placebo [24].

Esketamine, administered as an intranasal formulation in combination with an oral antidepressant, has demonstrated efficacy in multiple randomized controlled trials and is approved for TRD in several countries. In clinical studies, esketamine is typically administered at doses of 28-84 mg per session via a nasal spray device under medical supervision [21,24,25]. During the induction phase, dosing is generally performed twice weekly for the first four weeks, followed by a maintenance phase with once-weekly or once-every-two-weeks administration depending on clinical response and tolerability [21,24]. These regimens have been shown to produce significant reductions in depressive symptoms and lower relapse rates when used as adjunctive therapy [21,24].

Ketamine is a racemic mixture of R- and S-enantiomers, whereas esketamine is the isolated S-enantiomer. Both share a similar mechanism involving NMDAR antagonism and downstream enhancement of glutamatergic signaling via AMPAR activation and BDNF-mTORC1 pathways [12,23]. However, esketamine has a higher affinity for NMDAR and greater anesthetic potency [12], while emerging evidence suggests that R-ketamine may exert antidepressant effects through additional NMDAR-independent mechanisms [18]. These differences may contribute to variations in clinical efficacy, duration of effect, and side effect profiles.

Notably, the majority of clinical trials evaluating ketamine and esketamine in TRD have been conducted in adult populations, typically involving individuals aged 18 years and older, with limited data available in pediatric and adolescent groups [21-24].

Suicidal Ideation

Both ketamine and esketamine have demonstrated rapid reductions in suicidal ideation, with ketamine appearing particularly useful in acute psychiatric settings due to its ability to reduce suicidal thoughts within hours of administration [24]. Notably, the dosing used for the management of suicidal ideation is generally similar to that used for TRD, typically involving subanesthetic doses (e.g., 0.5 mg/kg IV infusion over 40 minutes), rather than distinct or higher dosing regimens [22,24]. It is, however, important to note that while ketamine has demonstrated antidepressant effects that may persist for several days to weeks in some patients, its anti-suicidal effects are generally more transient, often lasting hours to days. Accordingly, ketamine should be considered an adjunct to comprehensive suicide risk management rather than a standalone intervention. Comprehensive suicide risk management typically includes close clinical monitoring, safety planning, restriction of access to means, optimization of pharmacotherapy, and appropriate psychotherapeutic interventions [4,22]. Within this framework, ketamine may provide a rapid short-term reduction in suicidal ideation, thereby creating a critical therapeutic window that allows for the initiation and stabilization of longer-term interventions [22]. However, given the limited evidence regarding sustained reduction in suicide attempts and long-term outcomes, its use should be integrated within a structured, multimodal treatment approach [4].

Pan et al. conducted a nationwide cohort study in the United States to examine changes in suicidality following ketamine prescription in patients with TRD [26]. They reported a significant reduction in suicidal ideation in both the short term (days 1-30) and longer term (days 1-270) after ketamine exposure; however, no significant reduction in suicide attempts was observed. The decrease in suicidal ideation was more pronounced among individuals aged 24 years or older, male patients, and White patients [26]. These findings should be interpreted with caution, as the study was observational in nature, lacked a randomized comparator, and may not be generalizable to broader or more diverse populations. Moreover, the observed reductions in suicidal ideation likely reflect the combined effect of ketamine alongside concurrent standard treatments, rather than a direct comparison with other antidepressants or comprehensive suicide risk management strategies. Therefore, while the study suggests a potential association between ketamine use and reduced suicidal ideation, it does not establish a definitive or sustained effect on suicide attempts or overall suicidality outcomes.

Alarmingly, the study conducted by Pan et al. also found an association between ketamine prescription and an increase in suicide attempt in the short-term (days 1-30) in patients aged 10 years to 24 years [26]. It is imperative to note that suicidal ideation and suicide attempts are related but distinct clinical constructs. While suicidal ideation reflects the presence of thoughts or a desire to end one’s life, suicide attempts represent the behavioral enactment of these thoughts and are influenced by additional factors such as impulsivity, access to means, comorbid substance use, and environmental stressors. Therefore, a reduction in suicidal ideation following ketamine treatment does not necessarily translate into a proportional decrease in suicide attempts, as behavioral outcomes are determined by a broader range of clinical and psychosocial variables [26].

Table 1 highlights various key points pertaining to the use of ketamine and esketamine in psychiatric practice.

**Table 1:** Comparison of ketamine and esketamine in psychiatric practice FDA: Food and Drug Administration; TRD: treatment-resistant depression; REMS: risk evaluation and mitigation strategy

Sr. No.	Feature	Ketamine	Esketamine
1.	Chemical form [12]	Racemic mixture (R-ketamine and S-ketamine)	S-enantiomer of ketamine
2.	Regulatory status [25]	Off-label use in the treatment of depression	FDA approved for TRD
3.	Route of administration [20-24]	Intravenous (most common); oral and intramuscular (off-label)	Intranasal
4.	Dosing [20,23,24]	0.5 mg/kg intravenous infusion over 40 minutes	Fixed dose (28-84 mg) nasal spray; regardless of body weight
5.	Onset of antidepressant effect [2,7,8]	Within hours	Within hours to days
6.	Indications [22,23]	TRD, acute suicidality (off-label)	TRD (along with oral antidepressants)
7.	Monitoring requirements [4]	Blood pressure, heart rate, mental status during infusion	REMS-based monitoring post administration
8.	Adverse effects [7,8]	Dissociation, hypertension, nausea, dizziness	Dissociation, hypertension, nausea, dizziness
9.	Potential for abuse [4,27]	Higher, due to wider availability and off-label use	Lower, due to controlled distribution
10.	Accessibility [8]	Variable	Higher cost, often dependent on insurance

Special Populations and Off-Label Considerations

Evidence for the use of ketamine and esketamine in adolescents, older adults, and individuals with comorbid psychiatric or medical conditions remains limited. Preliminary studies suggest potential benefit in adolescent TRD; however, data are insufficient to support routine use, and careful risk-benefit assessment is required [28]. In older adults and medically complex patients, caution is warranted due to increased susceptibility to cardiovascular and cognitive adverse effects.

Ketamine is most commonly administered as an IV infusion at subanesthetic doses, typically 0.5 mg/kg over 40 minutes, although alternative routes such as oral, intramuscular, and intranasal administration have been explored in off-label settings [4,22]. Esketamine, the S-enantiomer of ketamine, is administered intranasally under supervised clinical conditions at doses of 28-84 mg per session, usually twice weekly during the induction phase, followed by once-weekly or once-every-two-weeks maintenance dosing depending on clinical response [21,24,25].

The primary indication for esketamine is TRD in adults, administered in conjunction with an oral antidepressant, while ketamine is widely used off-label for TRD and acute suicidal ideation in controlled clinical settings [4,23]. Contraindications and precautions include uncontrolled hypertension, significant cardiovascular disease, history of psychotic disorders, and active substance use disorders, given the potential for exacerbation of symptoms and hemodynamic instability [4,27].

Ketamine has also been considered for off-label use in acute suicidal ideation because of its rapid onset of action, with evidence suggesting a reduction in suicidal thoughts within hours after a single subanesthetic IV infusion, most commonly 0.5 mg/kg over 40 minutes [22]. However, its use in this context should be approached cautiously and should not be considered a standalone anti-suicidal intervention. Available evidence supports short-term reduction in suicidal ideation, but data are insufficient to conclude that ketamine reduces suicide attempts or long-term suicide-related outcomes [22,26]. Therefore, when used for acute suicidality, ketamine should be administered only in controlled clinical settings with close monitoring of mental status, blood pressure, heart rate, dissociation, sedation, and emergence reactions [4,22]. It should be integrated with comprehensive suicide risk management, including continuous risk assessment, safety planning, restriction of access to lethal means, optimization of pharmacotherapy, and appropriate psychotherapeutic or crisis-focused interventions [4,22]. Additional caution is warranted in patients with uncontrolled hypertension, significant cardiovascular disease, active psychosis, or substance use disorders because of the risks of hemodynamic instability, perceptual disturbances, and misuse potential [4,27].

Both ketamine and esketamine are associated with a range of adverse effects, most commonly transient dissociation, dizziness, nausea, sedation, and increases in blood pressure and heart rate. These effects are typically short-lived but necessitate monitoring during and after administration. Concerns regarding potential for misuse, cognitive effects with repeated dosing, and long-term safety remain areas of ongoing investigation [27,29].

Off-label use of ketamine has also been explored in conditions such as bipolar depression and post-traumatic stress disorder; however, findings are mixed, and further controlled studies are needed before broad clinical adoption [28,30].

Controversies, challenges, and future directions

Despite promising results, several important controversies remain. First, the durability of antidepressant effects is uncertain, with many patients relapsing after discontinuation of therapy. In a randomized relapse-prevention trial, patients who discontinued esketamine and continued on oral antidepressant plus placebo experienced significantly higher relapse rates compared to those who continued esketamine treatment, with relapse risk reduced by 51% in patients with stable remission (hazard ratio (HR) = 0.49; 95% CI: 0.29-0.84) and by 70% in those with stable response (HR = 0.30; 95% CI: 0.16-0.55) [24]. These findings indicate a substantial risk of relapse following discontinuation and suggest that maintenance therapy may be required in many patients. Second, there is no consensus regarding optimal maintenance schedules or duration of treatment [29]. Clinically, these limitations underscore the need for structured long-term treatment strategies and careful patient selection when incorporating ketamine and esketamine into routine practice.

Additional challenges include the high cost of therapy leading to insurance dependence, limited availability of specialized treatment centers, and variability in clinical protocols across institutions [4,27]. These factors have important implications for healthcare accessibility and equity, particularly in resource-constrained settings, and highlight the need for standardized clinical guidelines to ensure safe and consistent use.

The increase noted in suicide attempts with ketamine prescription in individuals suffering from depression aged 10 years to 24 years, as studied by Pan et al., warrants special caution in this age group and further analysis [26]. This finding reinforces the importance of integrating ketamine use within comprehensive suicide risk management frameworks and emphasizes that ketamine should not be used as a standalone intervention in vulnerable populations.

It is pertinent to note that ethical concerns regarding commercialization, accessibility, and potential long-term dependence also require consideration and should be addressed in future studies. From a systems perspective, the requirement for supervised administration and monitoring further limits widespread implementation and necessitates appropriate infrastructure and training.

Future research should focus on identifying predictors of treatment response and clarifying optimal maintenance strategies. Biomarkers, neuroimaging findings, and genetic profiles may eventually help guide individualized treatment selection. Further studies are also needed to evaluate long-term safety, durability of response, and real-world effectiveness across diverse populations.

Future research should also assess and better understand differences in response to ketamine treatment among individuals from different geographic and demographic backgrounds, as current data in this regard remain limited. Such insights will be critical in ensuring equitable and personalized application of these therapies.

Novel glutamatergic compounds, including R-ketamine and other NMDA-modulating agents, are currently under investigation and may provide similar efficacy with fewer adverse effects [30]. Integration of ketamine and esketamine with other interventional psychiatry modalities, such as transcranial magnetic stimulation, may also represent a promising avenue for future care [30]. Collectively, these developments highlight the evolving role of glutamatergic therapies and their potential to reshape the management of TRD.

Limitations

This review has several limitations that should be considered. As a narrative review, it is subject to potential selection bias, as it does not employ a systematic methodology for study identification, screening, and inclusion. Additionally, the included studies demonstrate significant heterogeneity in terms of study design, sample size, patient populations, dosing regimens, routes of administration, and outcome measures, which limits direct comparability and synthesis of findings.

A substantial proportion of the available evidence is derived from short-term clinical trials, with relatively limited data on long-term safety, durability of response, and sustained functional outcomes. This reliance on short-term outcomes may overestimate the persistence of therapeutic benefits and does not fully capture relapse patterns or long-term adverse effects.

Furthermore, many clinical trials have been conducted in controlled research settings with carefully selected patient populations, which may not accurately reflect real-world clinical practice. As a result, the generalizability of these findings to broader and more diverse populations, including adolescents, older adults, and individuals with comorbid psychiatric or medical conditions, remains limited.

Finally, variability in reporting of adverse effects, differences in monitoring protocols, and potential publication bias may further influence the interpretation of the available evidence. These limitations underscore the need for more standardized, large-scale, and long-term studies to better define the role of ketamine and esketamine in routine clinical practice.

## Conclusions

Ketamine and esketamine represent a significant advancement in the management of depression, particularly in treatment-resistant cases, by offering rapid therapeutic effects through novel mechanisms. Their growing use, including intranasal formulations, has improved accessibility and structured delivery in clinical settings. However, challenges related to the durability of response, long-term safety, and optimal treatment strategies remain. Careful patient selection and monitoring are essential to ensure safe and effective use.

As the field continues to evolve, further research aimed at optimizing outcomes and improving safety will be critical. Ketamine and esketamine are likely to remain important components of modern approaches to difficult-to-treat mood disorders.
